# 3D‐Printed Protein Models as an Educational Tool in Biochemistry Outreach

**DOI:** 10.1002/bmb.70030

**Published:** 2026-01-22

**Authors:** Oliver Osborne, Siobhan Clennell, Shaun K. Bremner‐Hart

**Affiliations:** ^1^ School of Molecular Biosciences, College of Medical, Veterinary and Life Sciences University of Glasgow Glasgow UK; ^2^ Glasgow Gaelic Secondary School—Àrd‐sgoil Ghàidhlig Ghlaschu Glasgow UK

**Keywords:** 3D printing, model‐based learning, molecular models, molecular visualization, outreach, protein structure

## Abstract

The abstract and complex nature of molecular biology often presents significant challenges for students at all levels of study. Traditional teaching methods, such as the use of 2D diagrams, may not fully convey the intricacies of these topics, leading to difficulties in comprehension and engagement. This study aimed to introduce 3D‐printed and virtual protein models into a secondary school classroom to enhance students' understanding of protein structure. 3D models were designed using ChimeraX and were either 3D printed or hosted online as interactive virtual models. A PowerPoint presentation was used to introduce the concept of protein structure in a didactic manner. Next, students answered questions on worksheets using the protein models. These worksheets promoted inquiry‐based and self‐directed learning through research‐guided questions and challenges. Feedback revealed that students found the workshop innovative and engaging. All participants indicated that the 3D‐printed models enhanced their understanding of protein structure and expressed interest in future hands‐on workshops. These findings highlight the potential of modern, model‐based teaching approaches to improve comprehension of protein folding and structure.

## 1 Introduction

Biochemical concepts can be challenging for students due to their abstract nature and complex content, often leading to difficulties in comprehension [1, 2, 3]. Although biochemistry is fundamental to understanding the molecular basis of life, traditional teaching methods may fall short in effectively conveying its key principles. As a result, students frequently struggle to connect biochemical knowledge with real‐world applications, limiting their appreciation of its relevance in fields such as medicine, biotechnology, and pharmacology.

Students are typically introduced to protein form and function through two‐dimensional diagrams, which oversimplify key molecular biology concepts. Research has shown that the use of physical models can significantly enhance student understanding of macromolecular structures by providing a more tangible and interactive learning experience. These models, which can include cardboard cutouts, paper “origami” models, and 3D printed structures, have been demonstrated to improve student comprehension [4, 5, 6]. Previously, studies using 3D‐printed models of proteins have demonstrated improvements in students’ conceptual understanding, as evidenced by post‐exposure assessments [7]. Furthermore, using 3D models to explore DNA–protein interactions has been demonstrated to enhance engagement and improve conceptual understanding [8].

Inspiring young people to engage with science is critical to fostering creativity and encouraging diverse participation in science, technology, engineering, and maths (STEM) fields. Pedagogical research indicates that students struggle to understand concepts if they do not find learning enjoyable [9]. Therefore, implementing innovative teaching methods is essential to inspire student engagement and improve learning outcomes. Notably, the percentage of students achieving A–C grades in National 5 Biology in Scotland has declined by approximately 6% between 2019 and 2024 [10]. While this decline is likely multifactorial, enhancements in pedagogical approaches may help mitigate some of the contributing factors.

Protein form and function are key components of the Higher Biology curriculum, as outlined by the Scottish Qualifications Authority (SQA). In this module, students are introduced to protein biology, beginning with the formation of polypeptides through peptide bonds between amino acids. The module also covers protein folding, emphasizing the role of hydrogen bonds. Effectively teaching these foundational topics is essential to ensure that students grasp these core concepts and build a strong understanding of molecular biology [11]. However, the focus on polypeptide formation often comes at the expense of a deeper exploration of structural organization. As a result, the crucial relationship between protein structure and function is frequently overlooked, limiting students' ability to understand its broader biological significance.

To address this, we introduced physical 3D‐printed and virtual protein models to a high school biology class. The application of these strategies has been predominantly confined to undergraduate education, leaving their potential within secondary education underexplored [12]. Through an interactive workshop, we aimed to deepen students' understanding of protein structure and function by moving beyond the 2D representations typically found in textbooks. The introduction of physical models allowed learners to explore key concepts, including secondary structure elements, 3D tertiary structures, the lock‐and‐key enzyme mechanism, and the specificity of protein–protein interactions. Feedback from students and facilitators indicated enhanced engagement and improved comprehension, highlighting the potential of tactile and visual models to enrich molecular biology education at the secondary level.

## 2 Methods

### 2.1 3D Printed Protein Models

All 3D printed models were generated from existing data available from the Protein Data Bank (PDB) (Table 1). Models were designed using UCSF ChimeraX (version 1.8 [2024‐06‐10]) [25]. For printing, models were exported as .stl files. These files were then imported into Autodesk Netfabb 2025 to determine the optimal printing orientation. Each orientation of the models had distinct properties, such as the size of the supported area, the volume of supports, and the center of gravity height. The orientation was selected to minimize printing time and filament usage.

**TABLE 1:** Protein Data Bank (PDB) proteins and codes.

Model	PDB ID	References
α‐helix	1PPT	[13]
β‐sheet	1JY4	[14]
Amylase	6GXV	[15]
Apoptosome (cytochrome c and apoptotic protease‐activating factor 1)	3JBT	[16]
Collagen	1BKV	[17]
Glucose transporter 1 (GLUT1) with glucose analogue	4PYP	[18]
Green fluorescent protein (GFP)	1GFL	[41]
Hemoglobin	2HHB	[19]
Collagen prolyl 4‐hydroxylase	6EVN	[20]
Keratin	6EC0	[21]
P53 and DNA	1TUP	[22]
Titin	2RJM	[23]
Ubiquitin	1UBQ	[24]

**FIGURE 1:**
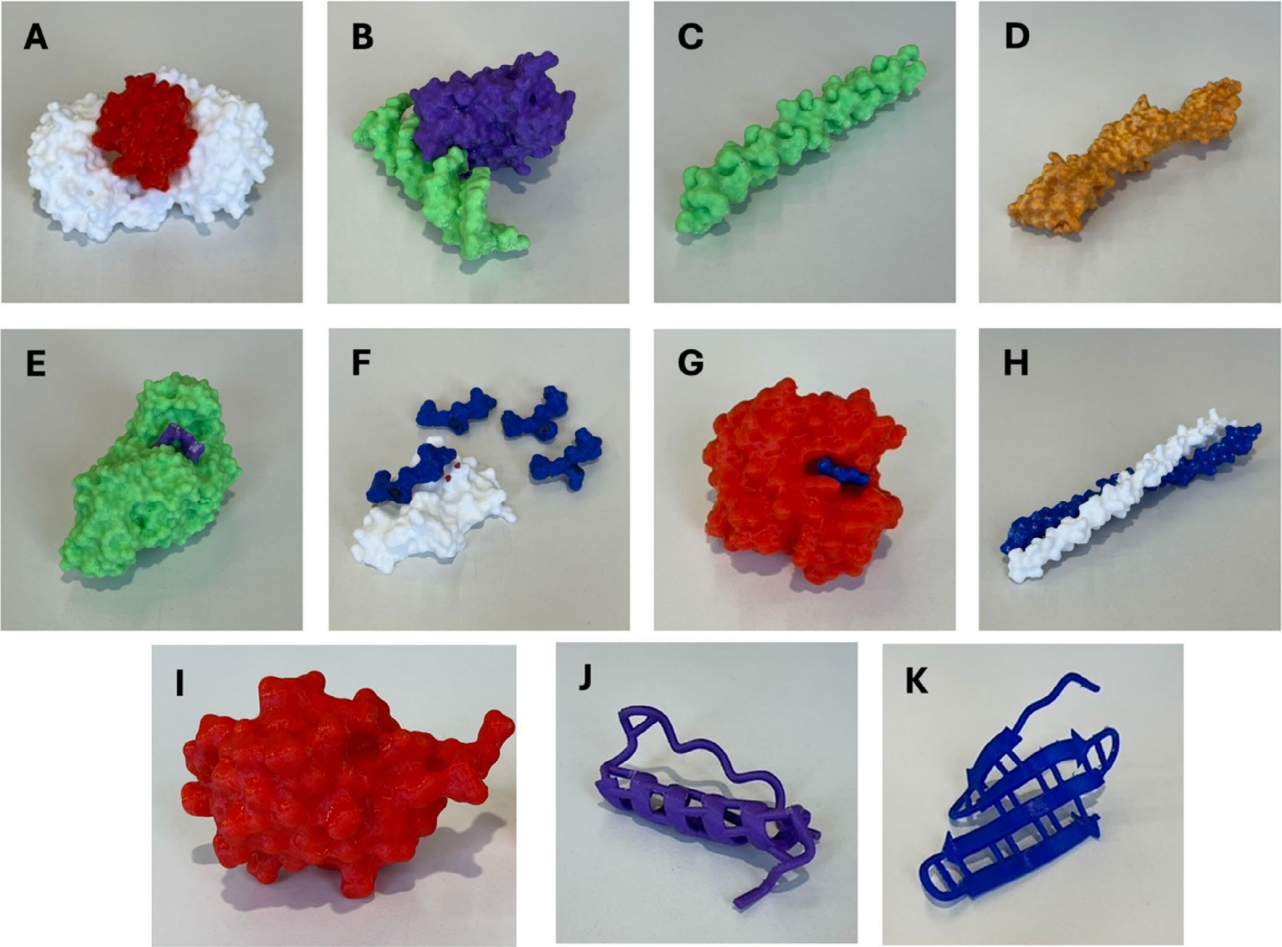
3D printed protein models. (A) Section of the apoptosome. (B) p53 (purple) and DNA (green). (C) Collagen. (D) Titin. (E) Amylase with carbohydrate analogue. (F) Hydroxylase (white) with native and mutated substrates (blue). (G) Hemoglobin (red) with haem (blue). (H) Keratin. (I) Ubiquitin. (J) α‐helix. (K) β‐sheet.

Finally, the protein models were uploaded to FlashPrint 5, which automatically generated the necessary supports. The software then sliced the models into horizontal layers for printing. The models were printed using a FlashForge Creator Pro 2 3D printer with polylactic acid (PLA) filament, at a nozzle temperature of 190°C and a heated bed set to 40°C. After printing, the support material was removed, and the models were finished by smoothing rough edges with a file (Figure 1).

### 2.2 Virtual Protein Models

To complement the 3D‐printed protein models, virtual models were also generated. Using ChimeraX, protein structures (Table 1) were exported as .glb files and uploaded to Sketchfab (https://sketchfab.com). Annotations were added to each model to provide details about the structure and function of the proteins. This additional information was included to help learners understand the biological significance and intricate features of each protein. The virtual models can be scaled and rotated, allowing for a thorough and interactive inspection of the proteins. This combination of physical and virtual models offered a multifaceted approach to studying protein structures, thereby enriching the educational experience.

### 2.3 Workshop Development

An active learning workshop was designed for Higher Biology students (aged 15/16, *n* = 10) at a high school in Scotland. The workshop was comprised of three distinct sections: a PowerPoint presentation, a worksheet activity incorporating 3D‐printed and virtual protein models, and an interactive quiz hosted on Mentimeter.

The PowerPoint presentation was delivered in a traditional didactic manner, covering the different levels of protein structure (primary, secondary, etc.) and the intramolecular forces that maintain these structures. During the discussion of secondary structures, students were provided with 3D‐printed models of an α‐helix and a β‐sheet (Figure 1). Time was allocated for students to inspect and ask questions about these models. Additionally, the presentation included information on globular and fibrous structures and the various functions of proteins, with a strong emphasis on the relationship between protein structure and function.

Next, students were given 3D printed proteins with an accompanying worksheet. The worksheets included images of the proteins from Sketchfab, a short description of the function of the protein, and four to five questions about the protein. The questions on the worksheets ranged from identifying the number of secondary structure elements that make up the protein to determining whether it resembles a fibrous or globular protein. Some worksheets also contained questions designed to foster independent research, such as “Use the internet to find other examples of where in the body keratin is found?” or “Use Google to investigate how the levels of titin in our muscles change as we age.”

Other worksheets focused on interacting with the models to identify cofactor or substrate binding sites. For example, the 3D‐printed model of hemoglobin was printed with the haem group separate from the polypeptide chain, and participants were tasked with identifying the haem binding site. Similarly, participants were able to identify substrate binding sites on models of amylase and demonstrate how keratin subunits interwound. To illustrate the lock‐and‐key mechanism, the model for collagen prolyl 4‐hydroxylase was developed along with its polypeptide substrate and alternative substrates with single amino acid substitutions. Participants were challenged to complete the following activity: “In the 3D protein model, there are 4 different substrates with different mutations altering their structure. Can you find the correct model that fits onto the hydroxylase enzyme?” Learners could identify the substrate binding cleft and identify which of the models was the native substrate.

Next, a Mentimeter quiz was designed to consolidate the information presented throughout the session. The questions were designed to align with the school curriculum, assessing whether learners had grasped the required information. Sample questions included “What name is given to a protein's primary structure?” (Answer: polypeptide) and “What type of bond is responsible for holding the secondary structure together?” (Answer: hydrogen bonds).

This study was reviewed and approved by the University of Glasgow's MVLS Ethics Committee (reference number: 200230381). In line with institutional practice for similar studies, informed consent was obtained through voluntary submission of the survey by participants. As the participants were under the age of 18, consent for their involvement was obtained from both the classroom teacher and the head teacher of the school. Responses were collected anonymously, and all identifying information was removed prior to data analysis. Participants were fully informed about the study's aims, what their involvement would entail, that participation was voluntary, and that they could withdraw at any time without any negative consequences prior to the workshop. This approach reflects the standard procedure for school‐based research within the university.

## 3 Results

**FIGURE 2:**
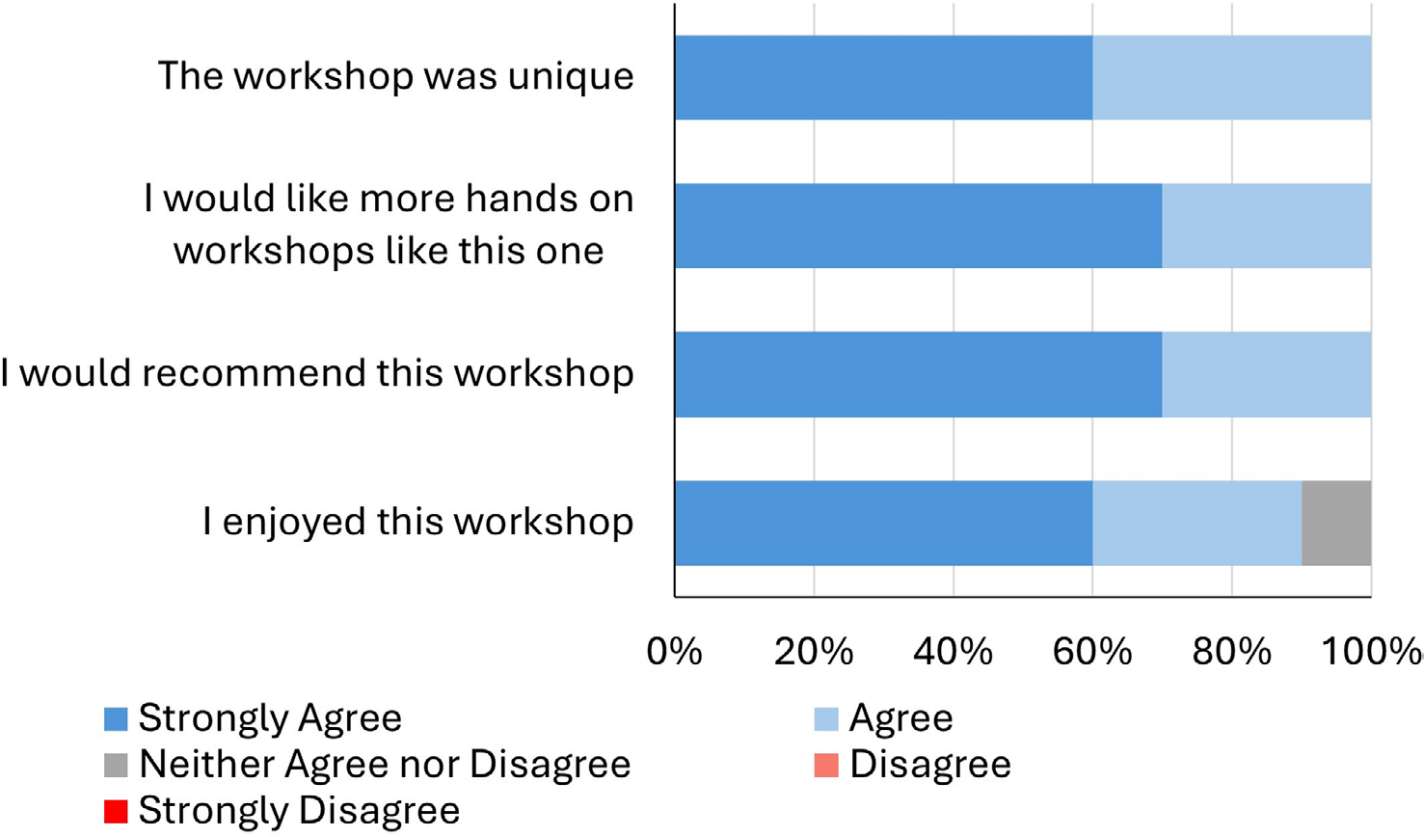
Post‐workshop student feedback summary. Participants were asked to complete an anonymous feedback form to collect their views on the workshop. *Note*: No participants selected disagree or strongly disagree.

Feedback and quiz scores showed that the workshop was both enjoyable and intellectually engaging for participants. The Mentimeter quiz was composed of nine questions featuring a mix of multiple‐choice and open‐text formats. The majority of participants answered all questions correctly, with success rates ranging from 70% to 100%. Following the workshop, the quantitative data from the questionnaires were analyzed. Of the 10 participating students, nine agreed or strongly agreed with the statement “I enjoyed this workshop,” indicating a high level of satisfaction among the participants. Additionally, 70% of the students strongly agreed that they would like more hands‐on workshops like this one in the future, highlighting the appeal of interactive learning methods (Figure 2). All participants indicated they would recommend the workshop to their peers and found it unique, suggesting that the workshop provided a distinctive and valuable learning experience.

**FIGURE 3:**
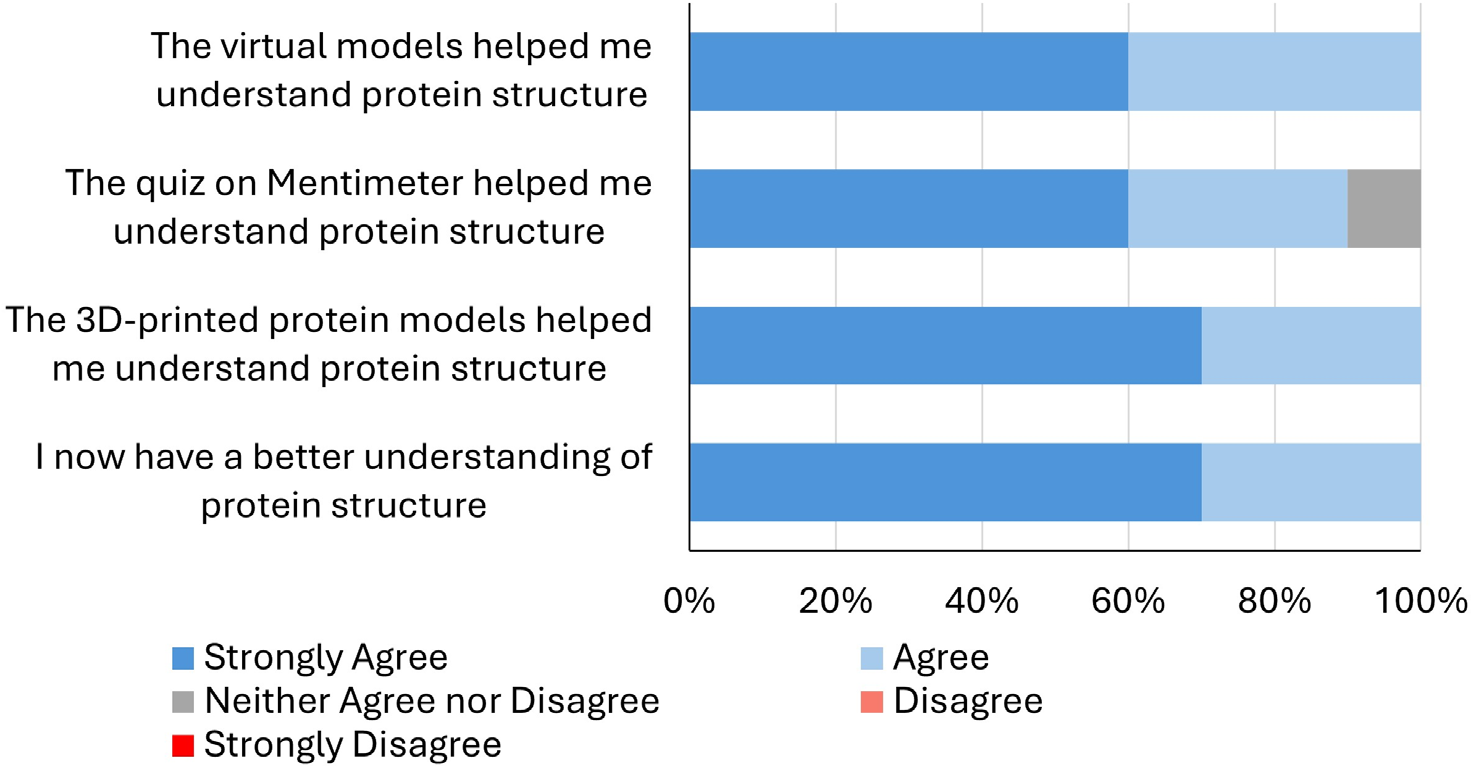
Assessment of how workshop components aided student understanding. Following the workshop, participants were asked to complete an anonymous feedback form evaluating different aspects of the session. *Note*: No participants selected disagree or strongly disagree.

Students were also asked to rate how informative they found the workshop. All students agreed that the 3D and virtual models were beneficial in aiding their understanding of protein structure (Figure 3). This feedback exemplifies the importance of using visual and tangible aids in teaching complex scientific concepts. While one student did not find the Mentimeter quiz particularly helpful, the majority of participants indicated that they had gained a better understanding of protein structure as a result of the workshop (Figure 3). This suggests that, despite some individual preferences, the overall approach was effective in enhancing students' comprehension.

Additionally, the questionnaire included open‐ended questions inviting students to reflect on their experience of the workshop. Students' use of adjectives “engaging,” “interesting,” and “innovative” to describe the workshop indicated that they perceived the session as a novel and enriching educational experience. The workshop results underscored the effectiveness of integrating interactive and hands‐on learning methods in enhancing students' comprehension of complex scientific concepts. The overwhelmingly positive feedback highlights the value of using 3D‐printed and virtual models to facilitate a deeper understanding of protein structures. The students' enthusiasm for more workshops of this nature suggests that such approaches can significantly boost interest in STEM subjects. Overall, the workshop successfully achieved its goal of sparking curiosity and improving understanding, demonstrating the potential of innovative educational tools in science education.

## 4 Facilitator Reflections

A secondary school educator reflected that teaching protein structure and function remains a persistent challenge, largely due to the abstract nature of the content and the depth of conceptual understanding it requires. They noted that students tend to underperform in assessments related to this topic compared to other areas of the curriculum, highlighting a gap in comprehension that may be attributed to the difficulty of visualizing molecular structures through conventional methods. The integration of 3D‐printed and virtual models in this workshop aimed to address this issue by transforming abstract concepts into concrete, manipulable representations. These models not only enhanced students' ability to appreciate the complex architecture of proteins but also supported a range of learning preferences. In particular, visual and kinaesthetic learners benefited from the opportunity to engage with the material in an active, tactile way. This approach emphasized the value of multimodal resources in demystifying challenging content and promoting deeper, more meaningful learning experiences.

The 3D protein models and worksheets were designed to incorporate multiple forms of media and models. Initially, there was some confusion about how the learners were to incorporate the physical models and the virtual models to answer the questions. Therefore, it would be beneficial to guide students through an example worksheet before allowing them to work independently. This approach would demonstrate to participants how to integrate information from the 3D‐printed models, virtual models, and independent research effectively. However, participants were generally engaged with the worksheets and were eager to receive new worksheets upon completing one.

Many of the learners opted to work through the worksheets in pairs. While this was not initially the plan, it provided an opportunity for learners to work collaboratively with their peers. This proved to be popular, and participants said that peer discussion about the tasks increased their confidence.

Based on prior teaching with only 2D representations, the addition of physical models substantially enhanced and immersed the learning experience. Illustrations often presented oversimplified and static depictions of protein structures, which may have contributed to misconceptions about the structure–function relationship. Similarly, while virtual models shown in animations or videos offered more flexibility, they still lacked the tactile and spatial engagement that physical models provided. The 3D‐printed models enabled dynamic, hands‐on manipulation, giving students a spatially grounded understanding of protein architecture such as active site–substrate interactions. Learners appeared more confident in identifying structural features like secondary structures and discussing functional implications when they could physically explore the models.

Learners demonstrated a keen interest in discovering proteins that were previously unfamiliar to them. Their enthusiasm for exploring new proteins not only demonstrated their eagerness to expand their knowledge but also highlighted the effectiveness of the workshop in stimulating intellectual curiosity. Informally, one learner noted that they had previously viewed proteins (specifically amylase) as unstructured blobs. The models used in this workshop demonstrated that proteins possess a defined structure, which can be visualized in great detail. This feedback suggested that textbook depictions of proteins may be overly simplistic, potentially leading to misconceptions about their complexity.

## 5 Discussion

This interactive workshop incorporated 3D‐printed and virtual models and received positive feedback from participants. Having already completed the protein form and function module with their teacher, the session was strategically scheduled to consolidate their prior knowledge. The workshop allowed students to revisit and reinforce what they had learned, while also introducing new insights and perspectives. Importantly, this intervention represents one of the few documented applications of 3D‐printed and virtual protein models within a secondary education context, addressing a gap in the literature that has largely focused on undergraduate cohorts. By integrating multiple learning modalities, the workshop effectively bridged the gap between theoretical knowledge and practical application, fostering a deeper understanding of protein structure and function.

Students were highly engaged during the introductory lecture, which covered the relationship between protein structure and function while consolidating prior learning. Given that attention span in lectures is often limited to about 15 min before students begin to lose focus [26], it was essential to keep this didactic section brief and interactive. The inclusion of secondary structure models during the presentation helped break up the monotony of traditional lecturing, effectively re‐engaging students and extending their attention span.

The protein worksheets effectively promoted inquiry‐based learning by integrating 3D protein models into the learning process. These models not only allowed students to visualize molecular structures but also served as interactive tools to explore complex biochemical concepts. The worksheets included research‐driven questions that encouraged students to engage with both the models and external resources, such as online databases and scientific literature. This approach fostered a deeper understanding by prompting students to actively seek out information, think critically, and make connections between theoretical knowledge and real‐world applications. By challenging students to apply their knowledge in a more self‐directed manner, the workshop sought to develop independent research and problem‐solving skills.

The Mentimeter questions were designed to align with the learning outcomes set by the awarding bodies, providing an authentic form of assessment. However, a key limitation of the study is the absence of a baseline measure of participants' prior knowledge. As the workshop followed standard teaching for this block, it was assumed that the activity would reinforce existing understanding. While this may be a reasonable assumption, without a pre‐intervention measure, it is difficult to determine the extent to which the workshop contributed to learning gains. Future iterations of this study would benefit from incorporating a pre‐session survey to assess participants' initial understanding [27]. This would allow for a more robust evaluation of the workshop's impact and help distinguish between knowledge gained during the session and that retained from prior instruction.

The use of 3D‐printed models offered a cost‐effective approach to enhancing biochemistry and molecular biology education. While an initial investment in a 3D printer is required, the cost of producing individual models remains relatively low. For example, the ubiquitin model used in this study, measuring 71.1 mm × 52.6 mm × 68.5 mm and required 40 g of PLA filament. Given that 1 kg of filament costs approximately £20, the cost per model is around £0.80. This affordability makes 3D printing a practical option for educational institutions seeking to expand their teaching resources without incurring substantial expenses. The positive feedback from the workshop participants further justifies the cost and time investment in 3D printing technology. The tactile and visual nature of these models provided a unique and engaging means of exploring complex biochemical concepts, which traditional teaching methods may not fully convey. Additionally, the ability to produce customized models tailored to specific educational objectives enables a more personalized learning experience. This flexibility accommodates diverse learning styles and preferences, making biochemistry and molecular biology more accessible and engaging for a wider range of students.

The use of multiple learning modalities was likely a key contributor to the success of this workshop. Physical 3D models, in particular, offer distinct advantages over virtual models. They have been shown to reduce the cognitive load associated with interpreting complex 2D representations by providing tangible, spatially accurate structures that students can manipulate [28, 29]. This hands‐on interaction supports spatial reasoning and helps students, especially those who struggle with abstract visualization, develop more accurate mental models of molecular structures.

In contrast, while virtual models offer dynamic features such as rotation, zoom, and embedded annotations, they can sometimes increase cognitive load, particularly when used in augmented reality environments [30]. Physical models, by comparison, simplify the learning experience by making abstract content more accessible and intuitive. Moreover, the combination of physical and digital tools caters to diverse learning preferences and has been shown to enhance students' intrinsic motivation to learn [31]. Thus, by combining the virtual and physical models, learners have an intellectually stimulating and enjoyable experience.

## 6 Implementation in Higher Education

The presented workshop could easily be adapted and implemented for students in higher education. Principally, this would involve increasing the complexity and depth of material. Such advanced content could focus on the conformational change induced by post‐translational modification. For example, protein phosphorylation causes substantial conformational changes that could be demonstrated using phosphorylated and unphosphorylated models. For example, serine 44 phosphorylation of DNA polymerase β causes large‐scale rearrangement of the protein backbone [32]. Similarly, kinase activation and the movement of the activation loop to regulate substrate access to the active site could be effectively demonstrated using 3D‐printed models [33].

Comparative structural biology could be demonstrated by developing models of protein orthologues and comparing the structures [34]. For example, the 20S proteasome varies in size and composition across different organisms [35]. Composition could be represented using differently colored filaments to illustrate the structural differences. Similarly, gene duplication and evolution could be demonstrated by the comparison of hemoglobin, myoglobin, and cytoglobin. In doing so, learners could explore the different protein structures and how haem is bound [36, 37, 38].

Biomedical applications could include how protein conformational changes and diseases. For example, how prion protein misfolding alters its conformation from a globular protein with high amounts of α‐helices to a disease‐causing conformation with a higher proportion of β‐strands [39].

In this study, models were constructed using PLA filament, resulting in static and rigid structures. The use of an alternative filament, such as thermoplastic polyurethane (TPU), which is flexible and rubber‐like, could provide a more dynamic representation of molecular interactions. For instance, TPU could be employed to illustrate the induced‐fit model of enzyme‐substrate interactions. Additionally, previous studies have utilized magnets to simulate the formation of secondary and tertiary protein structures, allowing components to move and snap into place to represent the stabilizing bonds that support protein folding [40]. Thus, this workshop represents a highly adaptable and customisable teaching activity that can be seamlessly integrated into various higher education curricula.

## 7 Conclusion

This study demonstrated the feasibility and pedagogical value of integrating 3D‐printed and virtual protein models into secondary education, addressing a gap in the literature where such approaches had been largely confined to undergraduate contexts. By aligning these tools with the Scottish Higher Biology curriculum, the intervention illustrated how tactile and visual resources could enhance conceptual understanding of protein structure and function while fostering engagement and motivation among learners. The findings suggested that cost‐effective, scalable innovations of this kind had the potential to transform molecular biology education at the secondary level, supporting the development of spatial reasoning skills and promoting early interest in biochemistry.

## Ethics Statement

Ethical approval for this study was gained from the University of Glasgow's MVLS ethics committee.

## Conflicts of Interest

The authors declare no conflicts of interest.

## Data Availability

The data that support the findings of this study are available on request from the corresponding author. The data are not publicly available due to privacy or ethical restrictions.
